# Maternally Perceived Barriers to and Facilitators of Establishing and Maintaining Tooth-Brushing Routines with Infants and Preschoolers

**DOI:** 10.3390/ijerph110706808

**Published:** 2014-07-02

**Authors:** Sarah Elison, Sarah Norgate, Lindsey Dugdill, Cynthia Pine

**Affiliations:** 1 Breaking Free Online Limited, 274 Deansgate, Manchester M3 4JB, UK; 2 School of Health Sciences, University of Salford, Allerton Building, Salford M6 6PU, UK; E-Mails: s.h.norgate@salford.ac.uk (S.N.); l.dugdill@salford.ac.uk (L.D.); 3 Barts and The London School of Medicine and Dentistry, Queen Mary University of London, London E1 2AD, UK; E-Mail: c.m.pine@qmul.ac.uk

**Keywords:** infants, preschoolers, tooth-brushing, routines, dental health, parental self-efficacy, self-efficacy scale, ecological model

## Abstract

Establishing effective toothbrushing routines using fluoridated toothpaste in infancy has been suggested as important to dental health throughout childhood and into adulthood. However, previous studies have revealed a number of potential barriers to, and facilitators of caregivers ability to establish early dyadic toothbrushing routines with pre-schoolers. However, as yet no qualitative research has been conducted to ascertain potential barriers and facilitators of the earliest dyadic toothbrushing in infancy, and nor has any previous research specifically focused on how novice mothers of first-born infants and preschoolers manage this task. This study therefore outlines findings from a qualitative interview study with first-time mothers of children aged 24–30 months (*n* = 16) exploring perceived barriers to and facilitators of early dyadic toothbrushing routines with infants and preschoolers. A number of key themes were identified from interview transcripts and an ‘ecological’ approach conceptualised maternally perceived barriers to and facilitators of dyadic toothbrushing. Proximal influences were found to be located within the caregiver-child relationship (‘micro-system’), including parental cognitions (e.g., PSE), parental behaviours (e.g., parenting practices) and infant and preschooler temperament and behaviours (e.g., tantrums). Distal factors were also identified as relevant to the establishment and maintenance of these routines, such as social support (‘exosystem’) and family history of tooth-brushing (‘chronosystem’).

## 1. Introduction

Establishing effective tooth-brushing routines using fluoridated toothpaste in infancy is considered to be one of the best ways to ensure a child’s dental health, as these behaviours, once established, endure throughout adulthood [1] providing lifelong protection against dental decay. There is now a growing body of published research, specifically qualitative research that has examined influences on the emergence of toothbrushing throughout childhood [2,3,4,5,6]. This research has identified that a number of influences may be associated with how dyadic tooth-brushing routines may be established, including child temperament and behaviour parental cognitions such as parental self-efficacy (PSE) [2,3,5]. 

However, most of the previously published studies have primarily included interviews with caregivers that have children who have had their full set of primary teeth for some time, so may not provide insights into influences on dyadic tooth-brushing from the point at which they are first established at the eruption of the first set of teeth. One exception here is a study by Hoeft *et al*. [6] involving children aged 3 months to 10 years. Although overall, the sample size was sizable for this topic, involving over 45 maternal caregivers, the focus was predominantly on caregivers rather than dyads as the unit of analysis. Given that children’s first primary teeth erupt at around age eight months, and the last primary teeth erupt at around the age of 24 months [7] is particularly during this developmental period that tooth-brushing routines need to be first established [8]. Further, understanding the developmental pathways through a focus on the unit of the dyad rather than either the individual caregiver or the child is important for understanding the barriers and facilitators to toothbrushing.

Despite the contributions made by the previous work exploring influences on dyadic tooth-brushing, no studies have specifically focused on dyads involving *novice caregivers and first-borns*. The inclusion of novice caregivers of first-borns in a qualitative study of their experiences of establishing toothbrushing as a dyadic process may be most informative as novice mothers may have had no previous experience of engaging in this parenting task. More generally, addressing novice caregivers is an important milestone in family life and the establishment of health practices.

Although previously published studies of dyadic tooth-brushing have not aimed to explore influence from *infancy* [2,3,4,5,6], they have fruitfully used qualitative interview methods to explore in detail potential influences on dyadic tooth-brushing with children who have developed beyond the period of infancy. Therefore, using a similar qualitative interview methodology may also provide unique insights into caregiver’s self-reported experiences of establishing tooth-brushing as a dyadic process from *infancy*, in dyads containing novice caregivers and first-borns.

Notably the previously published studies have included both *mothers and fathers* but in working towards an ‘ecology’ of the emergence of oral health behaviours it may be first advantageous to gain detailed insight into specifically *maternal* perceptions of their experiences of establishing and maintaining the routine especially in infancy, with mothers usually being principal caregiver at this point in development [9] in many cultures. The previous studies have included mothers and grandmothers [5], mothers and fathers [2,3], with only two including only mothers [4,6]. 

Maternally perceived influences on dyadic toothbrushing may be identified as acting as either barriers or facilitators to establishing dyadic toothbrushing and may be conceptualised by locating identified influences on relevant conceptual models. For example, in the past ecological models employed in public health research have explored influences on health behaviours, such as Stokols’ social ecological model [10,11] amongst others. However, in the context of examining influences from the *caregiver-infant dyad,* and specifically when examining influences on the emergence of toothbrushing as a dyadic process in infancy, what is being prioritized is the emergence of reciprocal caregiver-infant interaction during a health care task. Therefore, we argue that it is more appropriate to utilise an ecological model like Bronfenbrenner’s [10,11,12] that has been used extensively in infant developmental outcomes research, as opposed to the public health ecological models available in the literature.

**Figure 1:**
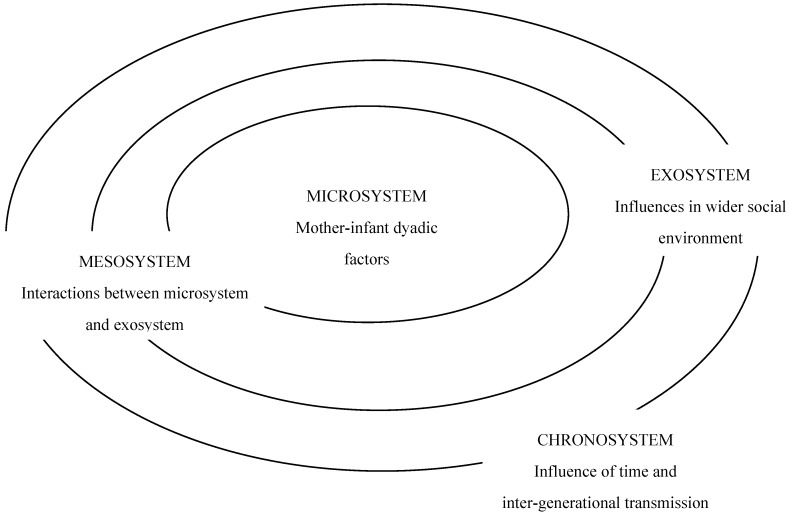
The Bronfenbrenner ecological model.

Ecological approaches like Bronfenbrenner’s [10,11,12] seek to describe the ways in which *human* development and behaviour interact with environmental factors. These environmental factors are conceptualised as lying at various levels, with interactions occurring between the various levels of influence. Such models identify both the immediate proximal influences, and also the more distal influences on child developmental outcomes, locating these within concentric, bi-directionally interacting levels. This means that influences lying at one level of the model may impact on and affect influences lying at other levels. At the core of the model is the notion that the individual interacts with the various levels of environmental influences to create an inter-relational process of development. Figure 1 depicts Bronfenbrenner’s ecological model and the levels of influence included within it. Such ecological conceptualisation of influences on dyadic tooth-brushing has never before been attempted within the published qualitative interview studies of dyadic tooth-brushing routines [2,3,4,5,6]. 

This study therefore aims to explore more specifically novice mother’s retrospective perceptions of influences on the emergence of tooth-brushing with fluoridated toothpaste as a dyadic process from infancy into the preschool period. Specifically how these influences may be perceived as barriers to, or facilitators of, the establishment of the behaviour when it is first established and then maintained as a routine through infancy are explored. Additionally, this new study has used Bronfenbrenner’s ecological model [12,13,14] to both inform interview item content within the qualitative interview schedule and also conceptualise themes identified from interview data. Mother’s reflections on their experiences of establishing tooth-brushing as a dyadic process are used to identify themes from interview data. These themes are used to suggest maternally perceived barriers to and facilitators of the establishment and maintenance of tooth-brushing, which are then located on the various levels of Bronfenbrenner’s ecological model in order to allow conceptualisation [12,13,14]. Guidelines around the use of thematic analysis in qualitative psychology research, are used to analyse data [15] are used and a 6-phase process is followed. This is to ensure that flexibility of the thematic analysis conducted does not undermine the theoretical and methodological rigour.

## 2. Experimental Section

### 2.1. Participants

Following ethical approval at institutional level, mothers were recruited from local child care services which were visited by the lead author (SE). Initially, mothers were approached and provided with study information before giving informed consent. As this was primarily an exploratory study, the only eligibility criteria were that participants were first-time mothers of infants and preschoolers aged between the ages of 24–30 months (*n* = 16) at the time data were collected, who had no previous experience of establishing dyadic tooth-brushing routines. All resided in one of two ‘districts’ in Greater Manchester that represent the worst rates of decayed, missing and filled teeth (DMFT) in under 5-year old children; Langworthy, Salford- 70%–79%, Ordsall, Salford- 60%–69%. For the purposes of this study, infants are defined as being aged 12 months and under and preschoolers 12 months to 36 months. Participants were offered the opportunity to be reimbursed for their time with a voucher that could be used in a number of well-known high street retail stores. Participants’ demographic data is summarized in Table 1.

### 2.2. Procedure

Following written consent, mothers were visited at home where interviews were conducted and lasted approximately 30 min. Interview schedules were unstructured and contained open ended questions, with topics for discussion being guided by potential influences on infant toothbrushing routines that were located across the levels of Bronfenbrenner’s ecological model [12,13,14]. 

**Table 1:** Demographic details of the sample.

Maternal age in years	Mean 30.66 (sd 3.53; range 22.83–35.00)
Infant age in years	Mean 2.22 (sd .16; range 1.92–2.50)
Infant gender	8 Female (50%), 8 male (50%)
Ethnicity	12 White-British (76%) 1 White-Irish (6%) 1 White-other (6%) 1 White/Afro-Caribbean (6%) 1 Pakistani (6%)
Marital status	8 Married (50%) 7 Cohabiting (44%) 1 Divorced (6%)
Current employment status	2 Full-time employment (13%) 6 Part-time employment (37%) 2 Part-time education (13%) 6 Full-time carers (37%)
Maternal employment type	7 Skilled (non-manual) (44%) 6 Unemployed/full-time carer (38%) 2 Partly skilled (12%) 1 Skilled (manual) (6%)
Educational record	8 Higher education (50%) (≥17 years in education) 8 Further education (50%) (14 years in education)

Topics questioned included exploration of current general childcare routines aspects of development through infancy, advice received about childcare, and how these issues related to dyadic toothbrushing. An example of a question is: “I’d like you to describe what sorts of things you do to take care of your child on a daily basis.” All face to face interviews were conducted in participant’s homes between March–September 2010 and then were transcribed verbatim. All transcripts and their themes and sub-themes were checked three times in order to check accuracy of coding and search for possible further themes and sub-themes that had not originally been identified. This process was continued until no new themes or sub-themes were identified from the data and theoretical saturation had been reached indicating no further data were required to be collected. 

Transcription was conducted by the researcher (SE) and transcription accuracy was checked in 20% of the audio files and their corresponding transcripts by a second researcher not associated with the study (TK). All transcripts were found to be accurate representations of audio data.

After all interviews were fully transcribed in Word, documents were imported into QSR NVivo 8 [16]. The main theoretical framework used to guide data analyses and identify themes was the Bronfenbrenner’s ecological model [12,13,14]. Barriers to, and facilitators of, tooth-brushing routines located at each of the four main levels of the model (microsystem, mesosystem, exosystem, chronosystem) were coded. Attempts were made to identify discrete, specific themes at each of these four levels that were associated with how well mothers managed to establish twice-daily dyadic tooth-brushing. 

Following data analyses, inter-rater reliability analyses were conducted by a second researcher (TK). An Intra-Class Coefficient (ICC) was generated to derive level of agreement of sub-themes assigned to 20% of the data collected by the first researcher (SE). The ICC coefficient generated for all sub-theme codings was 0.77 (*p* < 0.001), indicating overall substantial reliability across all sub-themes included in the analyses. Ethical approval for this study was granted on 26 April 2010 by the University of Salford Research Ethics Committee Ref: REP10/036. 

## 3. Results and Discussion

### 3.1. Findings

In general all mothers reported they brushed their infant or preschoolers teeth twice a day, usually in the morning after breakfast and in the evening before bedtime, and all mothers reported they used fluoridated toothpaste. They also reported that they were coping with dyadic toothbrushing. It is not known how long brushing sessions lasted within each dyad, although all mothers reported they felt their child’s teeth were cleaned effectively during toothbrushing sessions.

Overall, a total of five main themes were identified that were considered to be barriers to, and facilitators of, establishing and maintaining tooth-brushing routines with fluoridated toothpaste with infants and preschoolers. A number of sub-themes contained within the five main themes were also identified which were located across the levels of Bronfenbrenner’s ecological model [12,13,14]. These themes are reported below, and are ordered in a hierarchy from more proximal influences on tooth-brushing (those located within the mother-infant dyad within the ‘microsystem’ of Bronfenbrenner’s ecological model), to more distal influences on tooth-brushing (those located within the ‘mesosystem’, ‘exosystem’ and ‘chronosystem’ of Bronfenbrenner’s ecological model). Quotes representing each of the themes identified are also provided to illustrate how mothers perceived each as a barrier to or a facilitator of dyadic tooth-brushing routines.

**MICROSYSTEM**

**THEME** **1—Maternal cognitions**

‘Maternal cognitions’ are the attitudes and perceptions that mothers may have concerning their parenting role, which are known to have a significant impact on maternal behaviour and parenting practices. Mothers perceived a number of attitudes, or cognitions, pertaining to self-efficacy and their perceived level of control, as being associated with their ability to establish and maintain dyadic tooth-brushing routines.

(i) **Perceived maternal self-efficacy for tooth-brushing:** Almost a third of the mother’s interviewed (6/16) reported that they it was important to feel confident that they could establish dyadic tooth-brushing routines, which could be defined as ‘self-efficacy’, in order for them to successfully establish twice daily toothbrushing with their child. Some described perceiving the task of establishing dyadic tooth-brushing routines as challenging, but because they saw the task as important, they therefore felt confident they could, and should, establish the routines successfully. For example, Participant 8 says:
  *“…you’ve got to trust yourself a lot more than, like I was a bit ‘oh I don’t know what to do’. But actually you do know what to do.”*
(ii) **Perceived locus of control for tooth-brushing:** Three mothers reported that as a mother, they were the individual who controlled tooth-brushing, not their child. Despite non-compliant behaviours, they felt that they had enough authority to ensure they brushed their infant or preschooler’s teeth regularly and to an adequate level of hygiene. So for example, Participant 1 said:
  *“I’m the parent, she’s not the parent, so I need to make a decision on her behalf…”*
(iii) **Perceived outcome expectancies of establishing tooth-brushing routines:** Three mothers had positive ‘outcome expectations’ and reported that they saw tooth-brushing routines as valuable and necessary and something that could preserve their child’s dental health. Participant 2 described the possible outcomes if a parent fails to establish dyadic tooth-brushing routines:
  *“…I’ve heard a lot of horror stories about kids having to have their teeth pulled out and things like that. There’s absolutely no way at all I want to have that for (infant’s name)… ”*
(iv) **Perceived maternal stress:** Three mothers reported feelings of stress related to difficulties they experienced whilst attempting to establish dyadic tooth-brushing routines. One mother in particular reported that she suffered from Chronic Fatigue Syndrome and that this caused even routine parenting tasks to be tiring and stressful:
  *“I lose it. I try losing it behind closed doors. But ‘cos he knows I’m unwell, he kind of gets to a point when he gets frustrated ‘cos there’s stuff we can’t do. But I get brain tired, and I can’t cope ‘can I do this, I want to do that’. My brain shuts down.”*
  Participant 9
(v) **Maternal ability to remember to brush:** One mother also reported experiencing some difficulties in remembering to brush her child’s teeth, especially at night:
  *“I’m forgetful. I know you can’t really forget about it. When you’re thinking of everything else, you just forget.”*
  Participant 10

**MICROSYSTEM**

**THEME** **2—Parenting behaviours**

All the mothers interviewed reported using a number of parenting strategies to help them to overcome barriers to establishing and maintaining dyadic tooth-brushing. These barriers were largely associated with difficult, non-compliant infant and preschooler behaviours.

(i) **Initiating tooth-brushing early in infancy: **Initiating tooth-brushing as early as possible was reported by almost all mothers (13/16). Tooth-brushing routines were initiated either before the first tooth had erupted or as soon as this happened and was reported as being important in helping infants get used to tooth-brushing from as young an age as possible. One mother reported the mechanism by which initiating tooth-brushing as early as possible helped the facilitation of the routine:
  *“…you introduce them to the brush before they realise it’s something that they don’t like*.”
  Participant 9
(ii) **Allowing infant or preschooler to have a go at brushing their own teeth: **Another strategy employed by over half of mothers (9/16) to maximise compliance during tooth-brushing was allowing their infant or preschooler to have a go at brushing their own teeth during a tooth-brushing session. Participant 1 describes this process of turn-taking during tooth-brushing:
  *“…she gets to have a go, and then we rinse the toothbrush. And then I have another go, and she gets another go, and we rinse the tooth-brush and it goes on*.”
(iii) **Infant/preschooler modelling parent’s tooth-brushing behaviour:** Approximately half of mother’s interviewed (7/16) reported that they used a tooth-brushing technique in which they brushed their own teeth whilst allowing their infant or preschooler to observe whilst they were doing it. So for example, Participant 10 described:
  *“I stand her on the toilet and I brush mine …she does hers. And she copies. She copies a lot. So she does hers, and then I do mine, and then I say ‘let mummy do it’, and she lets me do hers for her.”.”*
(iv) **Creating a game out of a tooth-brushing session:** Another strategy, reported by approximately one-third of mothers (6/16) was turning tooth-brushing into a fun game (e.g., singing a song whilst brushing). Participant 15 describes how she used fun songs about tooth-brushing to make the activity enjoyable for her infant:
  *“I sing like a daft song, like that one on CBeebies [children’s television programme]. Or I do that song, you know the one in Grease…‘brusher, brusher, brusher’…”*
(v) **Disciplining infant/preschooler if non-compliant during tooth-brushing: **Approximately a third of mothers (6/16) reported that sometimes it was necessary to discipline their infant or preschooler (e.g., by withholding privileges) when they exhibited non-compliant behaviours during tooth-brushing. For example, Participant 8 describes how she withholds privileges like bedtime stories before having to resort to more punitive discipline:
  *“…just give him a few chances and then it’s like ‘do it or you’ll lose your stories’. But if it’s too bad then it’s like ‘we’ll count to 3 and then daddy’s going to hold you down and do it for you.”*
(vi) **Restraining infant/preschooler if physically non-compliant during tooth-brushing:** The more punitive strategy of physically restraining an infant or preschooler, to ensure tooth-brushing had been completed effectively, was reported by just over one third (6/16) of mothers. This technique was usually employed when infants displayed non-compliant behaviours in response to tooth-brushing (e.g., tantrums). Participant 14 describes how her husband would provide physical restraint in order to ensure their daughter had her teeth brushed properly:
  *“…he [husband] has to like hold her in a head lock and she just screams, it’s awful!”*
(vii) **Routinisation of tooth-brushing:** Routinisation of tooth-brushing, and the embedding of tooth-brushing into a wider repertoire of routine hygiene behaviours was also reported as being important to the maintenance of the behaviour by just under one-third of mothers (5/16). Participant 9 describes how she has embedded tooth-brushing onto a full repertoire of bed-time routine behaviours:
  *“…it was just kind of like part of bath, teeth, stories, and bed. It was just kind of the routine that we did it. And we still have the same routines now. Bath and teeth, stories and bed.”*
(iix) **Maternal perseverance with tooth-brushing when faced with difficulties:** A quarter of mothers (4/16) also reported that it was important to persevere in the face of difficulties when trying to establish and maintain dyadic tooth-brushing routines. These difficulties tended to be around difficult, non-compliant behaviours that disrupted tooth-brushing sessions. Participant 10 describes how she had to persevere with brushing her daughter’s teeth in order to get her used to the routine:
  *“Like don’t give in really. That is the main thing and I think she got used to brushing her teeth’ cos I stuck to it every day. It’s just persistence really. Like make sure you do it every day so they get used to it.”*
(ix) **Providing rewards for infant compliance during tooth-brushing: **One mother (Participant 9) also reported that it was important to provide rewards for her infant when they exhibited compliant behaviours during tooth-brushing. The use of this positive parenting strategy to encourage infant compliance during tooth-brushing was used by this mother in conjunction with other strategies such as withholding privileges, mainly bedtime stories:
  *Researcher. “So you use the story as a reward?”*
  *Participant. “He loves books. So…a lot of the ‘I don’t want to’ is dealt with by ‘that’s fine but then I don’t want to read a story for you’ ”*

**MICROSYSTEM**

**THEME** **3—Infant/Preschooler Behaviours**

Difficult, non-compliant behaviours were found to be a common barrier to tooth-brushing, being reported by almost all mother’s interviewed (14/16). As reported in the previous section, parents employed a number of strategies to overcome these difficult infant behaviours to enforce tooth-brushing.

(i) **Infant/preschooler wanting to brush themselves causes non-compliance during tooth-brushing:** The most common difficult, non-compliant behaviour that inhibited tooth-brushing was where the child wanted to/attempted to man-handle the tooth-brush and brush their own teeth, which was reported by half of mothers (8/16). Participant 14 describes how time consuming it sometimes was to give in to her daughter desire for autonomy during tooth-brushing:
  *“…just lately ‘cos she’s Miss Independent it’s really hard to like [brush teeth]. Like she won’t let me do them, and she’s strong. You can’t, like she has to brush them herself so it takes about 10 minutes. And she wants to do it…”*
(ii) **General dislike of tooth-brushing causes non-compliance: **General dislike of tooth-brushing was reported by approximately half of mothers (7/16) as being a barrier to twice-daily dyadic tooth-brushing. This general dislike was reported by some mothers as resulting in difficult, non-compliant behaviours such as tantrums. So for example, Participant 6 described how her son would physically struggle during tooth-brushing because he disliked it so much:
  “…he just struggles. Like I get the toothbrush in his mouth and I literally just go like, dead quick. But that’s as much as I can do, ‘cos he just like struggles about. Yeah, he’s a nightmare.”
(iii) **Closed mouth/refusal to open mouth inhibits tooth-brushing:** A less common, difficult, non-compliant behaviour was refusal to open the mouth which was reported by a quarter of mothers (4/16). This made inserting the tooth-brush into the infant’s or preschooler’s mouth more difficult, and would have inhibited further stages of tooth-brushing such as the removal of food debris. Some mother reported that they coped with this difficulty by trying to force the tooth-brush into their child’s mouth, like Participant 12 for example:
  *“…she would open her mouth, and sometimes she wouldn’t open her mouth. And I used to have to kind of force the brush gently.”*

However, some mothers reported within the ‘creating a game out of tooth-brushing’ sub-theme was the technique of making facial expressions and vocalisations that encouraged opening of the mouth. So for example, Participant 1 describes:

*“…doing the kind of ‘eee’ and ‘ahh’ has helped to at least get her mouth in the right position for me to get in there…”*

(iv) **Attempting to man-handle toothbrush prevents tooth-brushing: **Some mothers (3/16) reported that their infant or preschooler sometimes attempted to man-handle the tooth-brush by grabbing at it whilst their mother was trying to brush their teeth. This behaviour was reported by these mothers as acting as a barrier to successful completion of tooth-brushing sessions. In some cases this tooth-brush grabbing was due to infant and preschooler desires to brush their own teeth. However, in other cases it may have been that some were not grabbing at the tooth-brush because they wanted to brush themselves, but rather because they were just grabbing at a moving object as infants and preschoolers tend to. Or they may have been grabbing the tooth-brush in order to remove it from their mouth because of a dislike of tooth-brushing. So for example, Participant 1 describes that her daughter would try to remove the tooth-brush from her mouth when she had grown tired of having her teeth brushed:
  *“…when she’s had enough she’ll try taking the toothbrush away from me, so she won’t let me do it anymore.”*
(v) **Infant/preschooler sleeping prevents parent from brushing their teeth:** Some mothers (2/16) reported that if their infant or preschooler was sleeping they did not feel they could wake then in order to brush their teeth. This may have been due to parents being concerned about being able to get them to go back to sleep again, as Participant 2 describes:
  *“I’ve skipped brushing his teeth ‘cos he’s in the car, he’s falling asleep, so I’m like, ‘I’m not waking you up to brush your teeth’.”*
(vi) **Disliking toothpaste taste causes non-compliance during tooth-brushing: **Some mothers (2/16) also reported that their infant or preschooler sometimes did not like the taste of toothpaste and that this could disrupt tooth-brushing routines. This finding concurs with anecdotal evidence from dentists that sometimes young children find the taste of certain toothpastes to be too strong, as Participant 11 describes about her own son:
  *Participant. “It’s a bit hard not to use the children’s one ‘cos he doesn’t like the adult one.”*
  *Researcher. “Is that ‘cos of the taste?”*
  *Participant. “Yeah, it’s a bit too strong for him.”*

**MESOSYSTEM** **INFLUENCES ON DYADIC TOOTHBRUSHING ROUTINES**

**Main Theme** **4—SUPPORT AND ADVICE**

Almost all (13/16) mothers interviewed reported receiving some kind of support and advice either from friends and family or professionals about how they should go about establishing toothbrushing routines with their infant or preschooler and many reported that they received support from individuals such as co-parents or friends or relatives. 

**SUPPORT** **AND ADVICE—Sub-themes**

**(i)** **Support provided by co-parent when establishing tooth-brushing routines:**

This sub-theme although reported as lying within the ‘mesosystem’ of Bronfenbrenner’s ecological model could potentially be located within the ‘microsystem’. However, for the purposes of these analyses the ‘microsystem’ was conceptualised as any influences lying within the infant-*principal* caregiver (*i.e.*, infant-mother) dyad. Although co-parents (*i.e.*, fathers) provided support with tooth-brushing within the family home environment, mothers in this study had significantly greater care-giving responsibilities than co-parents. Therefore, support provided by co-parents was external from the infant-principal caregiver dyad and therefore this support was conceptualised as lying outside of the ‘microsystem’ in the ‘exosystem’.

Approximately half of mother’s (7/16) interviewed reported that they received support from their co-parent who in all cases was each mothers’ partner. Of those mothers who did discuss the level of support their co-parent provided, most of these (6/7) reported that their co-parent provided invaluable support during the process of establishing tooth-brushing routines with their infant. For example, some mothers reported that their co-parent often took control of tooth-brushing when their child exhibited difficult, non-compliant behaviours during tooth-brushing:

*“I have to wait for (husband) to come back and he does it…”*

Participant 14

**(ii)** **General social support with establishing tooth-brushing routines and coping with child-rearing:**

Approximately two-thirds of mothers (10/16) interviewed reported that the social support they received from friends and family had been important in determining how well they coped more generally with caring for their infant or preschooler. In particular, mothers reported that the support they received from mother and baby groups at their local Children’s Centre was invaluable in helping them feel more confident in their parenting role. For example:

*“…definitely get along to the groups, the support groups. You can make friendships that aren’t just important to you personally, but also for the benefit of your children. And I think it helps you to cope with your life better, just generally.”*

Participant 5

**EXOSYSTEM**

**THEME** **4—Support and Advice**

Almost all (13/16) mothers interviewed reported receiving some kind of advice about how they should go about establishing tooth-brushing routines with their infant and maintain these through to the preschooler period, and many reported that they received support from individuals such as co-parents or friends or relatives. Some of this was more general support in coping with their parenting role, with other support being more specific to tooth-brushing, such as the support co-parents provided.

(i) **Support provided by co-parent when establishing tooth-brushing routines: **This sub-theme, although reported as lying within the ‘exosystem’ of Bronfenbrenner’s ecological model, could potentially be located within the ‘microsystem’. However, for the purposes of these analyses the ‘microsystem’ was conceptualised as any factors lying within the infant-*principle caregiver* dyad. Although co-parents (*i.e.*, fathers) provided support with tooth-brushing within the family home environment, mothers in this study had significantly greater care-giving responsibilities than co-parents

Approximately half of mother’s (7/16) interviewed reported that they received support from their co-parent who in all cases was each mother’s partner. Of those mothers who did discuss the level of support their co-parent provided, most of these (6/7) reported that their co-parent provided invaluable support during the process of establishing tooth-brushing routines with their infant, such as Participant 4:

*Researcher. “And does your husband help out with things like tooth-brushing?”*

*Participant. “yeah, he does. And she’s [infant] fine with it.”*

(ii) **Professional advice received about tooth-brushing and establishing the routines:** Three-quarters of mothers (12/16) spoke about having received some kind of advice about infant dental health from healthcare professionals such as health visitors and dentists. However, this advice was reported to be minimal and mainly related to the age *when* tooth-brushing should be initiated. So for example, Participant 4’s dentist advised her to introduce her baby as soon as possible to allow her to get used to it:
  *Researcher. “Has the dentist given you any advice about how to care for (infant’s) teeth?”*
  *Participant. “I remember him saying just let her chew on the brush, like when she’s quite young…”*

Although only Participant 4 reported that she received this advice, starting brushing early was reported as a strategy by the majority of mothers (13/16). However, very little advice was received from professionals regarding *how* mothers should best go about establishing tooth-brushing routines, and no mothers reported that they had received advice about how difficult infant behaviours should be dealt with when attempting to establish and maintain tooth-brushing routines. Additionally, much of the advice mothers received about their infant’s dental health was about dietary habits rather than tooth-brushing. So for example, Participant 9 describes the advice she received in her weaning support group:

*“I went to the weaning thing and one of the things at the weaning was about dental. I don’t remember it being particularly effective in telling me what to do, possibly ‘cos we’d already started [tooth-brushing]. But, erm, I think we definitely got leaflets about what they should and shouldn’t be eating.”*

(iii) **Non-professional advice received about tooth-brushing and establishing the routines:** Some mothers (3/16) reported that they had received advice from family members and friends, although this advice was about parenting more generally, rather than being specifically about dental health and tooth-brushing routines. So, as Participant 3 describes how she and her friends with young children would support one another and swap advice:
  *“I’ve got friends that have children, so they would say how it was going and you would say how it* [tooth-brushing] *was going, and we’d give each other advice. If you’d gone through that already. You can share ideas.”*
(iv) **General social support with establishing tooth-brushing routines and coping with child-rearing: **Approximately two-thirds of mothers (10/16) interviewed reported that the social support they received from friends and family had been important in determining how well they coped more generally with caring for their infant. For example, Participant 5 described how important the parenting support groups she attended had been to her:
  *“…definitely get along to the groups, the support groups. You can make friendships that aren’t just important to you personally, but also for the benefit of your children. And I think it helps you to cope with your life better, just generally.”*

**CHRONOSYSTEM**

**THEME** **5—Family History**

Some mothers (3/16) reported that their own experiences of tooth-brushing with their parents, as an infant, influenced their approach to how they established and maintained tooth-brushing routines with their own infant. These mothers reported that because they had been encouraged to brush their teeth twice a day by their own parents, they perceived this behaviour to be ‘normal’ and expected, and were more likely to establish twice-daily tooth-brushing routines with their own infant. Participant 1 describes this well:

*“…for me it’s just the norm and expected to brush your teeth twice a day. Whereas I’ve spoken to people over past few years and they only brush their teeth once day which is very strange to me. So culturally it’s from childhood, definitely.”*

### 3.2. Discussion

This qualitative study sought to explore novice mother’s self-reported experiences, of establishing tooth-brushing with fluoridated toothpaste as a dyadic process with first-born infants and preschoolers aged between 24–30 months old. Identified barriers to, and facilitators of, dyadic tooth-brushing were located upon the Bronfenbrenner’s ecological model of infant development [12,13,14] in order to conceptualise how these factors are associated with the establishment of tooth-brushing routines. The findings from this qualitative interview study largely concur with those from the very few studies that have explored parent and infant related influences on infant dental health routines [2,3,17]. However, data presented provide a much greater depth of detail then data from previous studies. 

The principle factors found to be associated with how successfully tooth-brushing routines may be established were found within the ‘microsystem’ of Bronfenbrenner’s ecological model and were related to the mother-infant dyadic relationship. The mother-infant dyad has previously been found to be the most influential sphere of influence on development in the early years of life [12]. In the present study, dyadic influences on early tooth-brushing were located within the mother (maternal cognitions and parenting strategies used whilst attempting to establish the routines) and the infant (specifically infant behaviours, especially difficult, non-compliant infant behaviours). These influences were perceived by mothers as either barriers to or facilitators of the routine depending on whether they disrupted the routine or aided its execution, with some influences being seen as both a barrier and facilitator, depending on the context. So for example, the parenting behavior of allowing the infant or preschooler to take a turn at brushing their own teeth could be viewed as a barrier if this prevented the mother from being able to take control of the brush, but could also be a facilitator if this turn taking could be offered as a reward to the infant or preschooler following compliant behavior.

There were a number of maternal cognitions that appeared to be associated with how successfully they established tooth-brushing routines with their infant. Findings indicate that when mothers felt confident that they could establish the routines successfully (have high self-efficacy) they are more likely to experience success in establishing the routines. This finding concurs with those obtained in other studies exploring the relationship between parental self-efficacy and aspects of childcare including providing appropriate discipline [18] and eating routines [19]. Additionally, when mothers feel they are in control of their infant’s tooth-brushing routines (have an internal locus of control) and expect that their efforts to establish the routines will be successful in preserving their infant’s dental health (have positive outcome expectancies) they are also more likely to experience success. 

Parenting strategies used by mothers when establishing tooth-brushing routines included ‘positive parenting’ techniques such as using rewards and turning tooth-brushing into an enjoyable activity that infants wanted to engage in. Additionally, providing infants with the opportunity to become an active agent in the activity by allowing them to have some control over proceedings and engage in brushing their teeth themselves, was found to be helpful (in addition to mothers ensuring that they themselves brushed their infant’s teeth to an adequate level of dental hygiene). However, this finding is at odds with advice from dental experts that children under the age of 7-years should be closely supervised during tooth-brushing. This is due to the fact that young mouths are very delicate so tooth-brushes can potentially cause damage if children are left unsupervised. Additionally, it is not until middle childhood that children have the motor and cognitive skills required to brush their teeth to an adequate level of hygiene.

However, it would appear from this study that infants responded more positively to tooth-brushing when it was less of a ‘procedure’ that was carried out upon them by an adult, and more of an activity that they could claim ownership and self-control of (whilst the activity is being closely supervised by an adult). Most of the infants in the interview study were reported as wanting to try to brush their teeth themselves. This finding concurs with Erikson’s theory of human development [20] and newer revisions of the theory [21]. Between the ages of 1.5–3 years, infants are suggested to reach a stage of development characterised by the need for autonomy. 

In addition to infant’s desire for autonomy during tooth-brushing, mothers reported a number of other difficult infant behaviours that contributed to ‘battles in the bathroom’ around tooth-brushing. These ranged from general non-compliance, such as tantrums to more specific behaviours, such as refusal to open their mouth and trying to man-handle the toothbrush. These kinds of difficult, non-compliant behaviours are commonplace during the infant and pre-school years and were reported in both the Amin and Harrison [3] and Heubner and Riedy [2] dental health interview studies with parents. A recent study reported rates of difficult, non-compliant behaviours in approximately 10% of UK children without developmental delay [22]. 

Despite the non-compliant behaviours reported by the majority of mothers, tooth-brushing routines were reported to be in place with all the families in the study. Mothers had managed to overcome the impact of non-compliance through the use of a number of positive parenting behaviours and strategies. Providing education on these strategies would form an invaluable component of interventions to help parents establish tooth-brushing routines with infants. The specific strategies used by mothers included a number of positive parenting strategies such as turning tooth-brushing into a fun game, for example by using songs and games. This again concurs with the wealth of literature in the importance of play for a number of infant developmental outcomes and for learning [23,24]. 

The fact that the mothers interviewed had all been successful in establishing tooth-brushing routines could be attributed to the fact that although these mothers resided in socially deprived wards in Salford, they were found to be demographically atypical of the environment they were living in. Almost half of the mothers interviewed had been to college and over half had received higher education. Additionally, over half of mothers were either in some form of part-time or full-time paid employment and one mother was currently engaging in a training courses. This may mean that although these mothers were living in a socially deprived area they were generally well educated and also had social support at home in the form of a husband or partner (in all but one case mothers resided with a partner who was the father of their infant). These mothers may therefore have been able to access guidance around establishing dyadic toothbrushing routines, and given the educational attainments of this group of mothers, it could be that they were naturally conscientious and motivated women that approached their parenting role with the same conscientiousness and motivation they has approached their education. Additionally, most mothers saw the importance of maintaining dental health through toothbrushing with fluoridated toothpaste and had been encouraged to brush their own teeth as children by their own parents, so this may have contributed to their success in establishing the routine in their role as a parent given the suggested role of intergenerational transmission of effective oral health routines [25].

A potential limitation to the study is that all participants were self-selecting and so if motivated to participate in research, may also conceivably have been motivated and capable caregivers more generally. This may mean that as a group they may have coped well with establishing tooth-brushing with their child and therefore may not have experienced the full range of barriers and challenges that less motivated caregivers may encounter. Additionally, as was discovered from demographic data, were unrepresentative of the assumed population of Salford, although this kind of limitation is common in much health research. 

Additionally, the study sample was smaller than in the only two other similar interview studies that have been conducted that have explored barriers to, and facilitators of, tooth-brushing routines with pre-schoolers [2,3]. The Amin and Harrison [3] study included a larger sample size than in the present study with *n* = 19 parents (14 mothers, 5 fathers) and the sample size in the Hubner and Riedy [2] study was considerably larger than both the Amin & Harrison [3] study and the present study at *n* = 44. However, data in this study were collected by three researchers, which averages a total of *n* = 15 interviews per researcher. The potential sample for the current study was also smaller than the previously published studies [2,3] as the current study focussed on first-borns specifically, whereas the previous studies did not restrict recruitment of participants to caregivers of first-borns.

The kind of interview data collected in this study has been demonstrated to be invaluable in informing the development of psychometric scales [26] such as PSE scales [27]. Previous studies have collected information on the various barriers to, and facilitators of desired behaviours, such as parenting practices [28] in order to develop scales to measure PSE [29]. Such PSE scales have then be used to identify parents who may be at risk of having low PSE and have also been used to evaluate interventions intended to improve PSE [30]. Therefore, the qualitative data collected in this present interview study has been used to develop a PSE scale specifically designed to measure parents PSE in relation to establishing and maintaining twice-daily tooth-brushing routines with infants (reported elsewhere). It is hoped that this scale, can be used as a screening tool to identify parents who may be at risk of requiring support when attempting to establish and maintain twice-daily tooth-brushing routines with their infants.

Additionally, further work has been conducted by the study authors to examine more closely how developmental stage of infants and preschoolers may influence dyadic toothbrushing, specifically around aspects of psychomotor development and how these may either act as barriers and facilitators of the conduct of dyadic toothbrushing. This work, utilizing an observational methodology has found that as psychomotor development progresses between 12 and 24 months this may act as a barrier to parentally conducted toothbrushing and increased infant and preschool desire to enact self-toothbrushing. This observational work has also allowed collection of reliable data around length of time dyadic toothbrushing sessions lasts, as this data was not collected in the present qualitative interview study. His work has implications for anticipatory guidance for infant and preschooler toothbrushing and knowledge around tool use at this developmental stage and will be reported elsewhere. 

## 4. Conclusions

Using qualitative data generated by interviews with first time mothers of *first-born* infants and preschoolers, this study has described the various retrospectively perceived barriers to, and facilitators of, establishing twice-daily tooth-brushing routines in the early years and conceptualised these using Bronfenbrenner’s ecological model [12,13,14]. Specifically, these data have described a variety of challenging situations that mothers face when attempting to brush their infant’s teeth, particularly surrounding the difficult, non-compliant behaviours many infants exhibit, and how specific parenting practices may help caregivers overcome these. 
